# Retracted: Potassium channel ether à go-go1 is aberrantly expressed in human liposarcoma and promotes tumorigenesis

**DOI:** 10.1155/2021/4803896

**Published:** 2021-01-30

**Authors:** BioMed Research International

*BioMed Research International* has retracted the article titled “Potassium Channel Ether à go-go1 Is Aberrantly Expressed in Human Liposarcoma and Promotes Tumorigenesis” [1], due to an error in the reported sequences.

It was raised to our attention [2] that the recombinant adenovirus control (Ad5-Control-shRNA) sequence, CTA GGT GTT CTA GTC TGG ACT, that was reported to be non-targeting actually targets glycophorin C (GYPC). The authors also did not discuss their previous related research, from which they reused around 1,500 words [3].

The authors could not be contacted.
